# Assent and consent in adolescent research: teachers’ perspectives from a developing country

**DOI:** 10.1016/j.heliyon.2019.e03116

**Published:** 2020-01-02

**Authors:** Mahmoud A. Alomari, Nihaya A. Al-sheyab, Omar F. Khabour, Karem H. Alzoubi

**Affiliations:** a Department of Physical Education, Qatar University, Doha, 2713, Qatar; b Division of Physical Therapy, Department of Rehabilitation Sciences, Jordan University of Science and Technology, Irbid, 22110, Jordan; c Faculty of Nursing, Maternal and Child Health Department, Jordan University of Science and Technology, PO BOX 3030, Irbid, 22110, Jordan; d Department of Medical Laboratory Sciences, Jordan University of Science and Technology, Jordan; e Department of Clinical Pharmacy, Jordan University of Science and Technology, Irbid, Jordan

**Keywords:** Health profession, Public health, Evidence-based medicine, Social sciences, Education, Teachers prospective, Consent, Assent, Adolescent, Middle east

## Abstract

Teachers play a vital role in facilitating research in schools. However, teachers' views of informed consent/assent for children participation in research in the Middle East have not been investigated. In this study, focus group interviews were conducted to understand high-school teachers' perspectives toward adolescent assents and consents. The teachers indicated that parent consent is important and should be coupled with sufficient information about the research study. The teachers added that assent is most important for children above 13 years old. Conversely, the teachers believed that parent approval is only important for invasive (such as research involves blood withdrawal) but not simple procedures. Most importantly, for procedures that are considered simple, part of the teachers do not acknowledge the significance of parental approval, such as body weight, or beneficial, such as new treatment. The results indicate that some of the teachers’ views were consistent with proper conduction of pediatric research. However, other views were worrisome and might warrant further studies and actions. Risks related need to be assessed and policies needs to be developed in order to ensure the proper conduction of pediatric research.

## 1 Introduction

Child participation in research is usually achieved in two distinctive stages, parent/guardian permission, which is called parental consent then child approval, which is called assent [1, 2]. Child assent encompasses developing autonomy agreement to participate depending on developmental level, given parental approval had been obtained [3]. These ethical practices are to create suitable environment for making informed decision, thus protecting the child's welfare [4].

In developed countries, these ethical conducts are usually applied, in which children's rights are thoroughly preserved using comprehensive, up-to-date guidelines [5]. According to these guidelines, the child reserves the right to accept or reject participation in research, even after obtaining parental consent [5].

Conversely, ethical conduct of pediatric research in developing countries are scarce, due partially to, ignorance, lack of rules and regulations, and insufficient reinforcement [2, 6, 7]. A multicenter study in Egypt, showed inconsistent awareness and attitude about different aspects of ethical conduct in humans among faculty members from 4 medically-related colleges [8]. Alahmad et al., interviewed professionals from 12 medical disciplines from several Middle Eastern countries and found that these professionals believed that informed consent/assent process is crucial for medical studies that involve children [7, 9].

For Jordan, the Jordanian Food and Drug Administration governs clinical studies that include children or adolescents through the Jordanian Clinical Studies Law, which was amended in 2011 [10]. Although that law does not state additional requirements to conduct research on children, it is obligatory for every clinical study, including those that involve children, to obtain approvals of both the institutional review board of the institution where the study will be conducted and the institution of the researcher [11]. Moreover, an additional approval from the Jordanian Food and Drug Administration is required, if the study is a drug-related trial. In fact, the Clinical Studies Law is firmly reinforced and monitored through Jordan [12]. However, there are no special requirement of a formal workshop or training for investigators who propose to conduct studies on human subjects including children or adolescents [11].

In the Middle East, people younger than 15 years old are expected to reach 100,000,000 (>35% of the population) in 2025. The youth population in Jordan is projected to have 28% increase to be about 1.5 million (~20% of the population) [13]. Therefore, in such a region, pediatric research is particularly important. Schools are among the common as well as vital venues for conduction of pediatric research, namely those related to health promotion or epidemiology. Teachers, as well, are pivotal for conducting research in schools. Most importantly, they are “guardians” and “gatekeepers” that hold legal and social responsibilities. They can also be facilitators for research and be researchers of their own. Therefore, it is important for teachers to be aware of the research ethics, especially children consent and assent., especially in the recruitment process, as they are familiar with their students and know how to motivate and engage them [14, 15]. However, teachers’ views of informed consent/assent for children participation in research in the Middle East have not been investigated before. Given the difficulties of obtaining effective parental consent and child assent, it is very important to understand and explore teachers' perception and views. In the current study, focus group interviews were conducted to understand high-school teachers' views and perspective to wards assents and consents during children participation in research.

## 2 Method

### 2.1 Study approach and procedure

The study used a descriptive qualitative design aimed at exploring teachers' perception and views toward parental consent and child assent during paediatric research. The data was obtained during two focus groups, one for male and one for female high school teachers. Five schools were randomly selected –using a simple random technique-from a list of all public high schools in Irbid and Arramtha districts in Northern Jordan. Of the schools that were offered to participate in the study, the first two schools that showed interest in participation were chosen. Research that involved schoolchildren is common in Jordan. This involves both invasive and survey/questionnaire studies [16, 17, 18, 19, 20]. The researchers asked the principals to help in facilitating teachers' recruitment based on their availability and voluntary participation. The researchers asked the teachers about their past experiences with research requiring child assent and parental consent, which impacted the process of and selection of questions in the focus groups. The focus group method was chosen over any other method of data collection (i.e. interview) because the concept is relatively uncommon and being in a focus group tend to stimulate and encourage other teachers to share their experiences, if any, and perceptions about this important sensitive topic. Seven male and six female teachers, with age ranged 25–46 and 30–44 years old, respectively, agreed to participate in the focus groups. The focus groups were conducted in an unoccupied room using a round table setting. Only participating teachers and the researchers were in the room to ensure confidentiality. Ethical approval was obtained from the Institutional Research Committee of Jordan University of Science and Technology for all aspects of this study.

The study purpose and procedures were explained to the teachers, and permissions were obtained from all teachers to audio-record the focus group interview to be transcribed later. Subsequently, an open discussion, using probe questions to facilitate discussion, was initiated and continued until reaching mutual understating of each theme/question between the focus group members. Intergroup discussions and interactions were allowed and encouraged, however the researchers intervened when the interactions deviated away from the purpose of the study.

### 2.2 Questions

To facilitate a reliable and systematic collection of data, questions were predetermined and used as guidance for the interviews. One of the researcher led and moderated the interviews whereas another researcher was present to take notes and for helping in the discussion as needed. Moderation of the interview involved presentation of the questions, encouragement of participation form all subjects involved in the focus group, reframing, validation of point of views of participants via repeating, and questions expansion if needed, therefore ensuring credibility of obtained information [21]. The questions and responses were kept confidential.

The development of the subset questions was aided by consideration of the available literature in the area as well as the field experience of the researchers. The guide was designed with key questions that were grouped to be used for reference and as prompts if necessary. The researchers were to elicit conversations and discussions within the realm of consent and assent of adolescent participation in research.

The interviews were later transcribed by an independent researcher who was not present in the two focus groups. Initial analysis of transcribed data indicated a point of mutual understanding had been reached. The focus groups were discontinued at this stage.

## 3 Data analysis

Interviews of all focus groups were verbatim transcribed into Arabic, then back-translated in full to English. Then, researchers who conducted the focus groups validated the accuracy of the transcripts. A content-based thematic analysis approach was adopted, where two researchers independently read the transcripts as a whole. Statements that were deemed as important were marked and then, categorized. Thereafter, the translations and the original transcripts were confirmed via notes, categories, and themes exchange. Where there was a discrepancy or a controversy, issues were resolved via deliberation among researchers, which included additional data coding and re-coding up until final codes were identified and agreement was reached on themes.

## 4 Results and discussion

The study examined high-school teachers' views on informed consent and assents in paediatric research. As in Table 1, the analysis revealed that the questions presented during the focus group interviews yielded 3 main themes. These themes were consent and assent importance, consent and assent depth of information, and written versus verbal consent and assent in paediatric research.

**Table 1:** Themes and questions presented in the focus group interviews.

Theme 1:		Parent versus children approval to participate.

	Q: 1	Do you think that all research procedures and types of tests, require the approval of the child and parents?
	Q: 2	When do you think that only parental consent needed?
	Q: 3	What would you do if the parents agreed while the children rejected participation in research?
	Q: 4	What would you do if the children assented to while the parents refused participation in research?
	Q: 5	Do you think obtaining either the parents or child approval is enough to participate in the research?
Theme 2:		Information in the consent form

	Q: 1	What is the amount and depth of information that should be provided in the informed consent to parents and children?
	Q: 2	Would the details, types, and depth of the information be the same, regardless of child age?
Theme 3:		Verbal versus written consent and assent

	Q: 1	When written consent is required and sufficient?

Teachers are important assets to research. When a research is conducted in the school, the students participating in this research are the responsibility of the school administration, including the teachers. They are one of children “guardians”, to assure, first and foremost, the overall affairs of the students, especially health and autonomy. They are the “gatekeepers” to ensure that consent/assent is properly obtained from the parents and children. Additionally, the teachers are extremely essential in organizing the research activities. They can determine the appropriate strategies suitable for the students, teachers, and researchers to conduct the study, without disturbing the educational mission of the school. Strategies include recruitment, time and place, communicating with legal guardians, and attending to emergencies. On another note, teachers can be researchers in their own. One of the special advantages of teachers, they have access to large bodies of students, either in same school or other schools, which makes it easier to conduct research. Since they are in the field, they are excellent candidate to conduct research to examine a variety of questions and hypotheses to verify the efficacy of new educational strategies and teaching techniques. Therefore, it is important for them to understand and appreciate the ethical aspects of research, including assent and consent, to confirm this research is honest, reliable, trustworthy, and objective.

Some of the recruited teachers who took part in the study actually participated in research requiring them to make decisions related to assent/consent of children/adolescents. The authors, however, did not ask direct questions to the teachers about their knowledge in regards to research concept and types. However, their responses to the focus groups questions reflect limited understanding of various types of research. Additionally, some of the teachers’ responses and views in regards to the need for written consent in research involving invasive procedures reflect some understanding of the possible risks involved in certain procedures.

Views of some teacher were in consistence with proper conduction of paediatric research. Yet, views of other teachers were worrisome and might warrant for further studies and actions. In fact, social, cultural, education, religion, law and codes of conduct impact ethics behaviors in every country. The current findings are of special interest and importance, since in the Middle East, behavioural choices, including ethical practices, are governed via a complicated interface between sociocultural, religious, and individual factors [7]. In the Middle East including Jordan, the religion that is predominant is Islam. Thus, the age definition of children could be diverse compared to the one adopted by other countries [22]. "Fatwa", which is an Islamic clergy-issued legal statement, was shown to be essential for developing sentiments related to suitable ethical behaviors [7, 23]. In addition, guardian influence on children decision, even beyond legal adulthood age (i.e. 18 years) has been enforced by cultural, social, religious and educational factors [9]. Furthermore, few countries in the Middle East have guidelines for ethical behavior of research in pediatric populations [24, 25]. Allover, in the MENA region including Jordan, multiple factors impact consent of parents and assent of children. These factors include age of the child, perception of parents, religion, culture and education, and ethical guidelines adopted. For example, according to the Islamic codes and Arab cultural norms, parents hold special responsibilities toward their children, even after reaching adulthood. Therefore, they are expected to be liable and run the affairs of their children. Subsequently, children are expected to respect and comply with parent decisions. Accordingly, participating in paediatric research is influenced largely by parent discretion and can dominates the child and teacher opinions. Especially, if the research involves invasive procedures drug trial. The parent education level can also influence the decision of participation in paediatric research [26, 27].

Given the physiological differences, it is insufficient, unscientific, unacceptable, and most importantly, unethical to apply research findings in adults to children [1, 28]. Research on children investigate childhood development, disease aetiology, subsequently improving diagnosis, assessing, and treating diseases, thus promoting child health. Moreover, research in children can lead to innovative medical findings essential for improving the quality of paediatric healthcare. Indeed, this research must be high quality and adequately ethical. Therefore, paediatric research is extremely essential.

Informed consent is a crucial component of ethical conduct during research on human subjects [29]. Additionally, schools are common venue while teachers serve important role for recruitment during research in adolescents. However, teacher views of adolescent consent and assent are not known in developing countries, namely the Middle East and Jordan.

### 4.1 Theme 1: parent versus children approval to participate

The teachers were asked: "Do you think that all research procedures and types of tests, require the approval of the child and parents?" All teachers agreed upon several aspects related to obtaining informed consent and assent during pediatric research. All participating teachers believed that parent approval is only important for invasive but not simple procedures, such as questionnaires. This is not in line with the recommendations of proper research [1], which might indicate that there is a need for emphasizing the importance of obtaining parental consent in all types of research [29].

The data also revealed slight discrepancy between teachers on the importance of obtaining parent consent for adolescent participating in research. Some indicated that parent consent is important for children participating in research. Other teachers however, emphasized that parental approval should be coupled with sufficient information about the research study. These results indicate that the teachers have some knowledge about the importance of parent approval for children involved in research, which is consistent with proper research conduct.

When asked "when do you think that only parental consent is essential?", the majority of the teachers responded that when the children are in 1^st^-6^th^ grades (ages 6-12 years-old). However, some added that parental approval can be sufficient for children participation in research, even if the child has refused to participate. The teachers explained that some of the study tests/treatments can be beneficial for the child, including obtaining vital measurement or trying a new or an expensive medical treatment. However, parental consent is important for all children up to 18 years old. Additionally, children of all ages should not be coerced to participate in research especially if the parents are not present, such as in school setting [29]. Furthermore, unless incapable, the child should comprehend and assent to participating in research even if the parents are present [29].

In a response to a subsequent question, teachers indicated that they would try to persuade the child, when the child rejects participation after parental approval. They also said that many students do not participate because they would be worried about privacy of the information. Therefore, the teachers would persuade the adolescents by assuring the confidentiality of the information. Furthermore, the teachers would persuade the adolescents even further, if the study tests/treatments are deemed beneficial for the adolescent.

*"I would try to convince the students to participate in the study, but if he/she continue to refuse, I wouldn't compel the student to participate"*.

*"Assuring and confirming information confidentiality, can persuade the student to participate in the study"*.

*"I would try to persuade the student to participate, especially if the study is beneficial"*.

Conversely, the majority of the teachers in the girls' schools would coerce adolescents to participate in the study, if perceived beneficial for them. According these teachers, the adolescents, especially the younger ones don't understand the importance of research. Additionally, they also emphasized they might force the students to participate when research involve medical treatment/procedure that can be beneficial for the student. However, some of the teachers said they would respect the students' choice. Though coercing the students and responsibility might be contradictory, coercing stems from the sense of responsibility. The teachers coerce the students to participate because research is deemed beneficial thus; the teachers think they are helping the students to take advantage of a “special” opportunity. However, while respecting the students' choice is desirable, forcing the student to participate is not acceptable research conduct, even after obtaining parent approval. Especially, the parents would not be present during research.

*"I would force the girls to participate, even if they rejected, especially the parents approved participation"*.

*"The students are too little and don't understand what good and bad for them"*.

All teachers in the two focus groups said that assent for children under 13 years old is not essential, as long as the parents consented to the study procedures. The teachers said that children under 13 years old are incapable of understanding the procedures, benefits, and risks of the study, thus cannot make appropriate decisions.

*"Providing sensitive information during filling questionnaires during research in children requires parental consent"*.

However, some students can comprehend various study aspects and make decisions. Therefore, obtaining assents from younger children need to be sought if possible to comply with the international laws and regulations of the proper conduct pf pediatric research practice [3, 30].

The teachers also added that assent is needed for children 13–18 years old, because younger children cannot comprehend the details of a study, thus cannot decide for their own. However, some of the younger children can comprehend study procedures and make decisions. Additionally, forcing children against their well might compromise study results [1, 29]. Therefore, researchers, with the help of teachers, should try to get the children assents to the study as much as possible. Moreover, the researchers and teachers should avoid forcing the adolescent students to participate in studies, especially the parents are not present during the study [31]. Forcing the subjects to participate clearly contradicts the autonomy principle and respect of the study participants (PMID: 28599638). In addition, parental engagement in the informed consent process of children research is required unless the IRB waived such requirements [32].

In a subsequent question, the teachers responded that whether or not parent or child approval is sufficient to participate in research, depends on the procedure.

*"Parents should give consent to procedures because they are aware of the child health, benefits, and can provide more accurate information"*.

Studies requiring accurate information or are deemed beneficial, parental approval is sufficient. However, if the procedures are simple, such as weight and height, then child assent is sufficient. Usually, all studies require both consents and assents [5], therefore, these views require attention from investigators during research. Educational workshops are needed to modify these views to avoid violation of proper conduct of research.

*"Providing sensitive information during filling questionnaires during research in children requires parental consent"*.

### 4.2 Theme 2: depth of information in the consent form

All teachers, agreed upon the importance of sufficient and simple information to parents and children, thus making informed decision. They also added that this information should be provided in a pamphlet or brochure to students and parents.

*“a pamphlet would be a great way to inform parents and students about the study and will help them decide better”*.

*“Younger children won't understand all details of study…simple information is enough for them”*.

These views are in accordance with international guidelines for informed consents. These guidelines indicate that the informed consent should be simple yet informative while assuring confidentiality, freedom of participation, and right to knowing the results [6, 29, 33, 34]. Additionally, the benefits, risks, and procedures of the study should be thoroughly explained. The teachers' views are consistent with these guidelines and indicate that they are aware of proper research conduct [6, 29, 33, 34].

However, the teachers continued that this information should not be as detailed or written for children younger than 13 years old, suggesting that general information is sufficient. While complex information might be overwhelming for you younger children, international guidelines suggest some information must be given to the children, however it should be simplified [7, 33, 34].

### 4.3 Theme 3: verbal versus written consent and assent

When asked, all teachers equally agreed on the importance of obtaining written consent and assent for invasive procedures such as blood withdrawal. However, verbal consent and assent for simple procedures, such as questionnaires, are sufficient, according to the teachers of both genders.

“If the research involves blood extraction, then written consent in needed for sure”.

*“Whenever the study is only a survey and doesn't require physical contact with the students, verbal consent would be enough, I guess”*.

While this might be sufficient in most circumstances, obtaining written consent is highly recommended, especially when children are involved. Written consent is a documentation of the study details and proof participation approval. Therefore, researchers and clinicians should always opt to obtain written consents and assents as much as possible [7, 29, 33, 34].

## 5 Conclusion

The study shows that the teachers are aware of some of the proper procedures for obtaining informed consents and assents during research in adolescent with some discrepancy between male and female teacher views. One aspect is the importance of obtaining informed consent and assent. The results show that the teachers have some views that are not consistent with proper conduct of research, however, these views have some controversies about paediatric research practice worldwide. In fact, some teachers might force the children to participate in research as long they deemed the study beneficial for the child. Researchers conducting studies in high schools should adjust for these views. Additionally, parties involved in high school research should devote thoughtful efforts to educate high schools teachers on proper ways of obtaining consents and assents. Finally, risks related need to be assessed and policies needs to be developed in order to ensure the proper conduct of pediatric research. It is worth mentioning that the focus group size and number are small. Therefore, future studies with larger sample size and number are needed to confirm the results and verify current speculations.

## Declarations

### Author contribution statement

Mahmoud Alomari, Nihaya Al-sheyab: Conceived and designed the experiments; Performed the experiments; Analyzed and interpreted the data; Contributed reagents, materials, analysis tools or data; Wrote the paper.

Omar Khabour, Karem Alzoubi: Conceived and designed the experiments; Contributed reagents, materials, analysis tools or data; Wrote the paper.

### Funding statement

This work was supported by grant # 5R25TW010026-02 from the Fogarty International Center of the U.S. National Institutes of Health.

### Competing interest statement

The authors declare no conflict of interest.

### Additional information

No additional information is available for this paper.
